# Systemic Action of Inflammatory Mediators in Patients with Essential Hypertension and Diastolic Chronic Heart Failure: A Clinical Pathophysiological Study

**DOI:** 10.3390/pathophysiology27010005

**Published:** 2020-12-12

**Authors:** Anton V. Barsukov, Alla Yu. Seidova, Ksenia A. Shcherbakova, Marina S. Black, Alexander E. Korovin, Leonid P. Churilov, Dmitry V. Tovpeko

**Affiliations:** 1 S.M. Kirov Military Medical Academy, St. Petersburg 194044, Russia; ksu2204@yandex.ru (K.A.S.); selmarina07@rambler.ru (M.S.B.); korsyrik@mail.ru (A.E.K.); tovpeko.dmitry@gmail.com (D.V.T.); 2 Medical-Sanitary Unit of the Ministry of Internal Affairs of the Russian Federation in St. Petersburg and Leningrad Region, St. Petersburg 191015, Russia; allo4ka1989@bk.ru; 3 Saint Petersburg State University, St. Petersburg 199034, Russia; elpach@mail.ru; 4 Saint Petersburg Research Institute of Phthisiopulmonology, St. Petersburg 194064, Russia

**Keywords:** essential hypertension, chronic heart failure, preserved ejection fraction, diastolic dysfunction, proinflammatory status, correlation analysis, sex differences

## Abstract

The aim of this research was to correlate indicators of proinflammatory status and the structural/functional characteristics of the cardiovascular system comparatively in male and female patients with essential hypertension (EH) complicated by diastolic chronic heart failure (CHF) with preserved left ventricular ejection fraction (LVEF). The study included 104 middle-aged patients (55 males (M) and 49 females (F)) with first- or second-degree EH complicated by CHF with preserved LVEF. They all belonged to the low functional class of CHF, with LVEF ≥50%, first- or second-degree of LV diastolic dysfunction (LVDD), LV hypertrophy (LVH), and dilatation of the left atrium (LA) with a sinus rhythm and N-terminal brain natriuretic peptide >125 pg/mL. Serum levels of C-reactive protein (CRP), tumor necrosis factor alpha (TNF-α), and interleukin-6 (IL-6) were measured. To identify the relationship between the proinflammatory pattern and cardiovascular parameters, Spearman’s rank correlation coefficients were determined. M had markedly higher levels of CRP, TNF-α, and IL-6 compared to F. However, all the mean values corresponded to the reference range. Significant direct associations of CRP level with the LV mass index (LVMI), relative wall thickness (RWT), LA volume index (LAVI), E/e’ ratio, and systolic and diastolic blood pressure (SBP, DBP) existed in both M and F, as well as negative correlations of CRP with LVDD parameter e’ and distance covered in a 6 min walk test. M and F had a positive association between IL-6 and LVMI, LAVI, E/e’ ratio, SBP, RWT, and DBP, as well as strong negative associations between IL-6 and e’ and distance passed in 6 min in each group. Significant direct correlations existed between serum TNF-α level and LVMI, RWT, LAVI, E/e’, SBP, and DBP both in M and F. Furthermore, there were negative relationships of TNF-α level with e’ and the distance covered for the 6 min walk. This study demonstrated a close relationship between the blood levels of proinflammatory autacoids and indicators of EH, exercise tolerance, LVH, LVDD, and LA enlargement, regardless of the patient’s sex. Compared to female patients, male patients had stronger correlations of CRP, TNF-α, and IL-6 levels with indicators of LVDD degree.

## 1. Introduction

Essential hypertension (EH) plays a key role in the pathogenesis of chronic heart failure (CHF) with preserved left ventricular ejection fraction (LVEF). This form of CHF makes up 30–55% of all cases of circulatory failure cases [1,2,3]. Patients with chronic heart failure with preserved LVEF usually have a set of concomitant diseases (obesity, type 2 diabetes mellitus, renal dysfunction, chronic obstructive pulmonary disease, and so on). This form of CHF affects predominantly female patients. A noncardiovascular death scenario in CHF with preserved LVEF is more common than in CHF with low contractility of this heart chamber [4]. Over the last two decades, the problem of excessive systemic action of inflammatory autacoids on the cardiovascular system has been actively discussed. Acute excessive action of inflammatory autacoids is involved in pathogenesis in all forms of circulatory shock. However, chronic systemic excess of proinflammatory bioregulators is also a pathogenic and disturbing systemic neuroendocrine regulation [5]. The systemic equivalent of low-intensity inflammation partakes in the pathogenesis of many cardiovascular diseases and syndromes, including EH, metabolic syndrome, atherosclerosis, left ventricular hypertrophy (LVH), atrial fibrillation, and acute and chronic heart failure [6,7,8,9,10]. It is known that the inflammatory pattern is more strongly represented in CHF with reduced LVEF compared to the conditionally polar form of heart failure. By decreasing the bioavailability of nitric oxide and the development of concentric remodeling, as well as myocardial hypertrophy, low intensive inflammation in the cardiovascular system promotes the formation of left ventricular diastolic dysfunction (LVDD), a frequent satellite of CHF with preserved LVEF [11]. Although the prevalence of CHF with preserved LVEF has sex differences, the comparison of proinflammatory status in male and female cases of CHF with preserved LV contractility in homogeneous groups (age and body-mass-index-matched, with similar comorbidities and severity of the underlying diseases) was never assessed, although the immune systems of men and women have obvious differences impacting the pathophysiology of many immunopathological disorders [12].

Thus, the aim of our study was to establish, taking sex into consideration, a correlation between indicators of proinflammatory status and the structural and functional characteristics of the cardiovascular system in individuals with essential arterial hypertension, complicated by diastolic CHF with preserved LVEF.

## 2. Materials and Methods

### 2.1. Object of the Study

**Figure 1:**
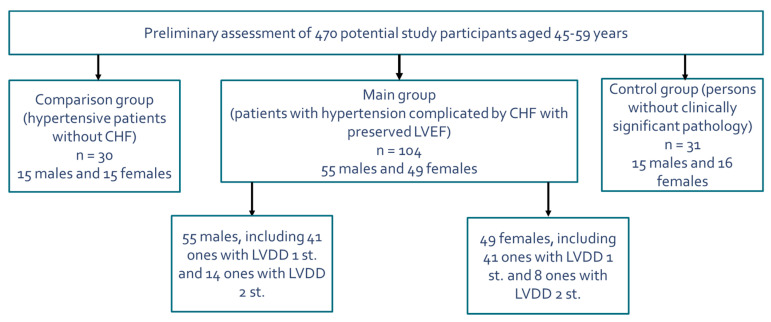
Scheme of the selection of study participants in the main group (combination of essential arterial hypertension and heart failure with preserved left ventricular ejection fraction). Abbreviations: CHF: chronic heart failure, LVEF: left ventricular ejection fraction, LVDD: left ventricular diastolic dysfunction.

The research was conducted at the clinical base of the Department of Hospital Therapy of the Military Medical Academy. At the prescreening, 470 units of medical documentation (outpatient records, medical records) of subjects aged 45–59 years were studied, as a result of which 104 patients (55 males and 49 females) suffering from EH and CHF with preserved LV systolic function were selected for the main group: 30 people (15 males and 15 females) with EH but without CHF were selected as the comparison group, and 31 people (15 males and 16 females) without hypertension and other clinically significant pathologies were selected as the control group. The current article provides a comparative analysis of data only within the main group of study participants. Thus, we evaluated the cohort of 104 specially selected patients (55 males and 49 females) with first- or second-degree EH complicated by CHF with preserved LV systolic function. A scheme of the selection of study participants in the main group is shown in Figure 1.

The individuals of the main group were selected according to the design of the research, representing a fairly homogeneous sample. Males and females were initially comparable in age, body mass index (BMI), level of systolic (SBP) and diastolic (DBP) blood pressure, heart rate (HR) at rest, and severity of CHF. The diagnoses of EH and CHF with preserved LVEF were established according to the current recommendations of domestic and European boards of experts [13,14,15,16]. The cohort included only patients with a clinical picture corresponding to a low functional class of CHF (first or second according to the NYHA classification), a LVEF equal to or greater than 50%, with a sinus rhythm, and plasma content of the N-terminal brain natriuretic peptide (NT-proBNP) >125 pg/mL. The level of NT-proBNP is determined by the method of solid-phase enzyme immunoassay with Cobas H 232 Kit (Roche Diagnostics GmbH, Mannheim, Germany). The functional CHF class was confirmed by the results of the 6 min walk test. The notion of CHF with preserved LVEF confirmed by the presence of the following criteria recommended by the European Society of Cardiology (2016) experts: presence of LVH (proven by LV mass index (LVMI >115 (M) or >95 (F) g/m^2^)), dilatation of the left atrium (LA volume index >34 mL/m^2^), and diastolic LV disturbances [16]. All individuals had first- or second-degree LV diastolic dysfunction (LVDD). The following exclusion criteria were considered: third-degree EH, secondary arterial hypertension, history of myocardial infarction, noncoronary myocardial diseases, valvular heart diseases, atrial fibrillation and other clinically significant arrhythmias, chronic kidney disease of the fifth stage, any clinically significant pulmonary diseases, thyroid dysfunction, acute inflammatory diseases or exacerbations of chronic diseases 2 weeks prior to screening day, as well as oncological diseases. Patients with the inability to ensure proper quality of echocardiography or the inability to assess diastolic LV function in sufficient capacity were not included in the study.

### 2.2. Laboratory Methods

The serum levels of the C-reactive protein (CRP), tumor necrosis factor alpha (TNF-α), and interleukin-6 (IL-6) were examined at 8–9 a.m. in fasting patients selected after the assessment of the inclusion/exclusion criteria. The CRP level in peripheral venous blood was measured by enzyme immunoassay with Sapphire-400 facility (Hirose Electronic System Co., Ltd., Tokyo, Japan), considering the normal level of less than 5.0 mg/L. The serum levels of TNF-α and IL-6 were assessed by means of a solid-phase enzyme-linked immunosorbent assay using highly sensitive test systems (Vector-Best, Novosibirsk, Russia). The normal range of TNF-α and IL-6 content was 0–6 pg/mL and 6–10 pg/mL, respectively. Biochemical blood analysis was performed with facility Spectrum (Abbott, Abbott Park, IL, USA). All patients underwent fasted blood sampling to determine the blood levels of cholesterol, triglycerides, high-density lipoprotein (HDL) cholesterol fraction, low-density lipoprotein (LDL) cholesterol fraction, glucose, uric acid, urea, creatinine, and potassium.

### 2.3. Instrumental Methods

Standard twelve-lead electrocardiograms were recorded with the ECG-9812 electrocardiograph (Medinova Industrial Co., Ltd., Shenzhen, China). Transthoracic echocardiography was performed according to standard procedures with the Philips EPIQ 7 ultrasound system (Philips Healthcare, Best, The Netherlands). Locating was performed at the chordal level of the mitral valve, directly below the free ends of its valves. Echocardiographic indices were evaluated in five consequent cardiac cycles with the calculation of mean values. In this position, the LV internal diastolic dimension (LVIDD, mm), the LV internal systolic dimension (LVISD, mm), the interventricular septum thickness in diastole (IVS, mm), and the posterior wall thickness of the LV in diastole (PWT, mm) were examined. LV end-systolic volume (LVESV, mL), LV end-diastolic volume (LVEDV, mL), and LVEF were determined from the apical four-chamber and two-chamber positions by the disk method or by the modified Simpson’s method [17]. The LV mass index (LVMI, g/m^2^) was determined as a ratio of LV mass (LVM, g) to the body surface area (S, m^2^) using the following formula:

LVMI = LVM/S(1)

The LVM was calculated by the following cubic formula recommended by ASE experts (1986) [18]:

LVM, g = 0.8 × (1.04 × ((LVIDD + PWT + IVS)^3^ − LVIDD^3^) + 0.6 (2)

Left ventricular geometry was estimated in accordance with the recommendations of the American Society of Echocardiography and the European Association of Cardiovascular Imaging (ASE/EACI, 2015) [19], which took into account the definition of LVMI and the relative wall thickness (RWT) of the LV. The RWT calculated by the following formula (ASE/EACI, 2015):

RWT = (PWT + IVS)/LVIDD(3)

**Figure 2:**
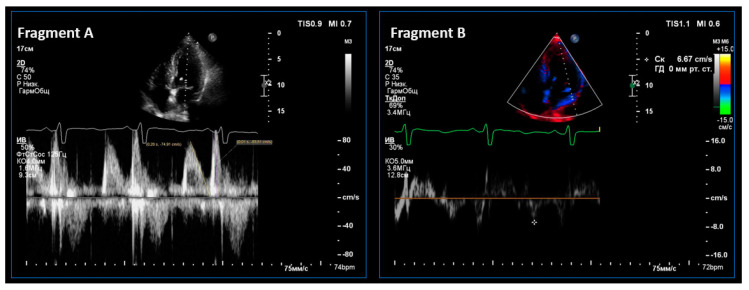
An episode of echocardiography in Participant A. (male, 55 years old). In the Doppler mode (Fragment A), transmitral blood flow is shown with an assessment of Peaks E and A. In the tissue, the Doppler mode and the fibrous ring of the mitral valve movement are shown in the left ventricle early-filling phase.

LV diastolic function was examined in the mode of tissue Doppler echocardiography. The average peak tissue velocity of early diastolic displacement of the septal and lateral parts of the mitral valve ring (e’, cm/s) was estimated, and the E/e’ index was calculated to establish the left ventricular filling pressure [16]. An episode of ultrasound assessment of left ventricular diastolic function is shown in Figure 2.

The left atrium volume (LAV, mL) measured by means of the area-length method in the B-mode using the formula:

LAV = 8/3π × A_1_ × A_2_/d (4)

where A_1_ is the area of the LA in the 4-chamber section, A_2_ is the area of the LA in the 2-chamber section, and d is the smallest size of the LA in the 2- or 4-chamber section (apical–basal).

In accordance with the recommendations of ASE/EACI experts [20,21], the LA volume measured by the above-mentioned method was indexed by the body area (mL/m^2^). The normal LA volume index (LAVI) for the examined individuals considered equal to or less than 34 mL/m^2^.

### 2.4. Statistical Analysis

Statistical data analysis was performed using the Statistica software package for Microsoft Windows (StatSoft, Inc., Tulsa, OK, USA; software version 10.0.1011.0). Normal distribution was verified by the Kolmogorov–Smirnov method with the Lilliefors correction. The group differences were estimated by determining the value of the Student’s *t*-test and the relationship between the Pearson categorical variables (criterion χ^2^). To study the relationships between indicators that did not obey the law of normal distribution, we used the module of nonparametric statistics (nonparametric Mann–Whitney U test). A critical level of significance was considered to be *p* < 0.05. Spearman’s rank correlation coefficients were calculated in order to identify the relationship between the proinflammatory pattern and cardiovascular system parameters (office BP, exercise tolerance, echocardiography data). The correlation was considered to be weak for the coefficient r < 0.2; moderate for r = 0.2–0.49; strong for r ≥ 0.5.

## 3. Results

The baseline characteristics of the examined patients with EH complicated by CHF with preserved LVEF are given in Table 1.

According to our study, the level of each of the measured proinflammatory markers in the examined individuals corresponded to the reference range of values regardless of sex. Males and females had practically identical values of electrocardiographic indices—the Sokolow–Lyon index, the Cornell voltage and product, and the amplitude of the R wave in aVL—which corresponded to the general population norm. Based on ultrasound data, both the male and female groups demonstrated a moderate concentric LVH with a slight left atrium dilatation, borderline changes (upward) of the E/e’ indicator, and a decrease in the e’ indicator, confirming the presence nonsevere LVDD. Males and females had no significant differences in parameters such as: LVIDD, LVISD, LVEF, RWT, and LAVI (*p* > 0.05 for all parameters). LVMI and the E/e’ ratio in males proved to be significantly greater (*p* = 0.03) and the exponent e’ was significantly smaller (*p* = 0.009) than in females. Taking into account the design of the research, the data given below show the interrelations of proinflammatory pattern indicators with the structural and functional cardiovascular system parameters in patients with EH complicated by CHF with normal contractility of the LV. Table 2, Table 3 and Table 4 show statistically significant correlations between the level of CRP, TNF-α, and IL-6, with office values of BP, exercise tolerance, and echocardiography data.

Significant direct associations of the serum CRP level with LVMI, RWT, LAVI, E/e’ ratio, SBP, and DBP (*p* < 0.001 for all parameters), as well as negative connections of this proinflammatory factor with e’ (*p* < 0.001) and the traversed 6 min distance (*p* < 0.001) both in male and female patients with EH complicated by CHF with preserved LVEF, are shown in Table 2.

**Figure 3:**
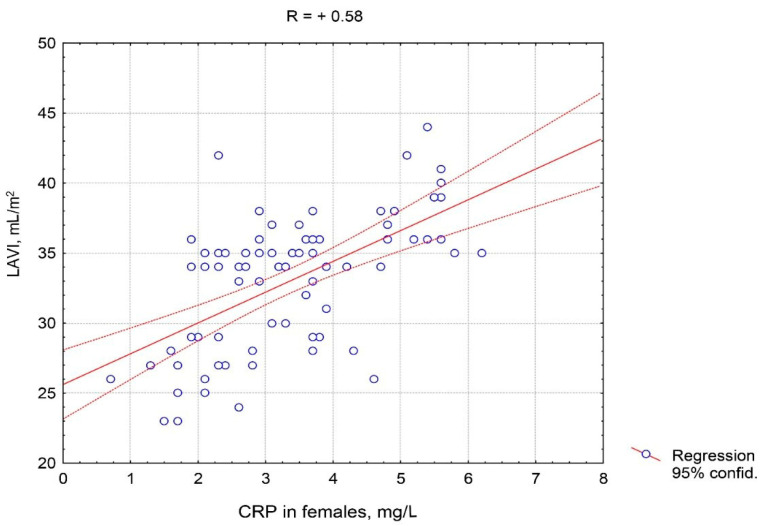
Correlative association of the C-reactive protein serum level and the left atrium volume index in females with hypertension complicated by CHF with preserved left ventricle ejection fraction. Abbreviations: CRP: C-reactive protein, LAVI: left atrium volume index.

**Figure 4:**
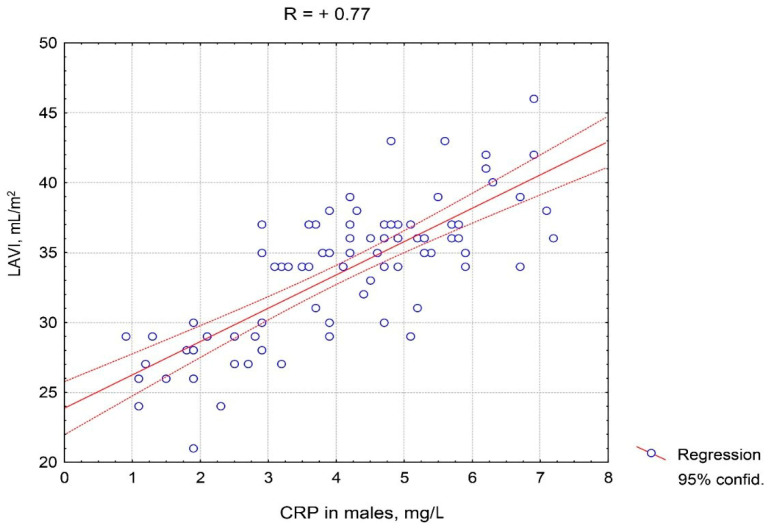
Correlative association of the C-reactive protein serum level and the left atrium volume index in males with hypertension complicated by CHF with preserved left ventricle ejection fraction. Abbreviations: CRP: C-reactive protein, LAVI: left atrium volume index.

It should be noted that the positive correlations of CRP with LAVI, E/e’ ratio, and SBP in males were stronger than in females. Figure 3 and Figure 4 depict the features of the association of CRP with LAVI regarding gender. A closer correlation of CRP with LAVI in males compared to females is clearly visible.

As indicated in Table 3, male and female patients with EH complicated by CHF with preserved LV systolic function characterized by significant positive associations of IL-6 with LVMI, LAVI, E/e’ ratio, SBP (*p* < 0.001 for all indicators in each group), RWT (for males: *p* < 0.001, for females: *p* = 0.003), and DBP (for males: *p* < 0.001, for females: *p* = 0.003). There were strong negative associations of IL-6 with e’ (*p* < 0.001) and distance passed in 6 min (*p* < 0.001). It is pertinent to note that a closer correlation of IL-6 with each of the indicators listed in the table was observed among males than females.

In Table 4, significant direct associations of serum TNF-α level with LVMI, RWT, LAVI, E/e’, systolic and diastolic BP (*p* < 0.001 for all indicators in each group) were observed both in male and female patients with EH complicated by CHF with preserved LV systolic function. There were also observed negative relationships of this proinflammatory factor with e’ (*p* < 0.001 for males and females) and with the 6 min walk distance covered (*p* < 0.001 for males and females).

**Figure 5:**
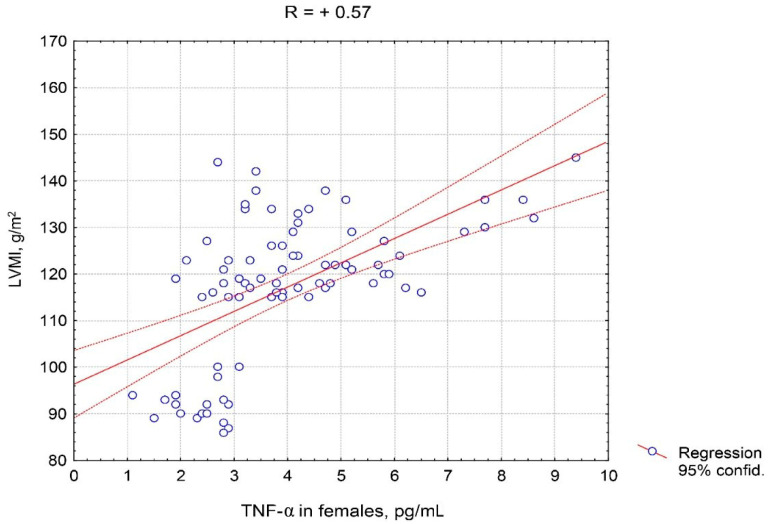
Correlative association of tumor necrosis factor alpha serum levels and left ventricular myocardial mass index in females with hypertension complicated by CHF with preserved left ventricular ejection fraction. Abbreviations: TNF-α: tumor necrosis factor alpha, LVMI: left ventricular myocardial mass index.

**Figure 6:**
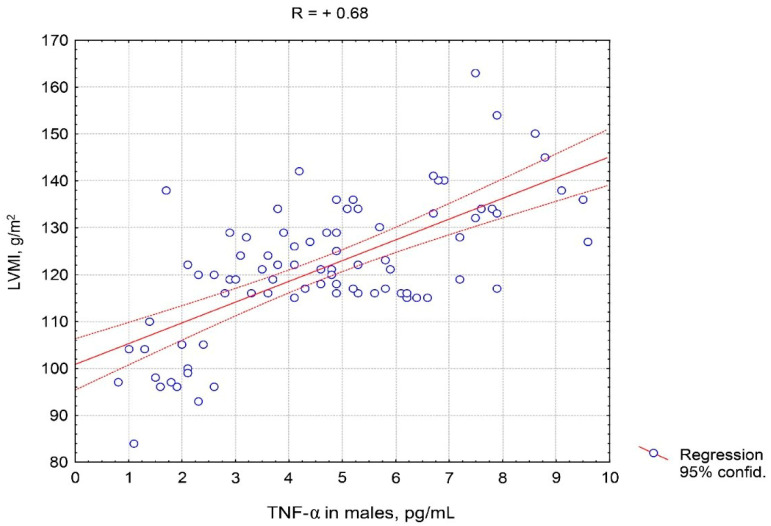
Correlative association of tumor necrosis factor alpha serum levels and left ventricular myocardial mass index in males with hypertension complicated by CHF with preserved left ventricular ejection fraction. Abbreviations: TNF-α: tumor necrosis factor alpha, LVMI: left ventricular myocardial mass index.

It should be noted that the correlation of TNF-α in males was stronger than in females with each of the indicators presented in Table 4, in particular with E/e’, e’, and the 6 min walk distance covered. Figure 5 and Figure 6 show the features of the association between TNF-α serum level and LVMI, taking into account the patient’s sex. The closer and practically linear relationship of TNF-α with this ultrasound parameter was observed in males compared to females.

## 4. Discussion

Many studies have proposed that the sex distribution of patients with EH and CHF is approximately equal. At the same time, among those who have heart failure with preserved LVEF, women prevail. It is seen more clearly in elderly populations [22]. The formulated paradigm about the importance of subclinical systemic action of immune and inflammatory autacoids in chronic arterial hypertension and heart failure dictates the need for a detailed study of these links of pathogenesis in order to optimize therapeutic solutions in clinical practice.

Immunoinflammatory activation with excessive systemic action of cytokines and other related autacoids plays an important role in the pathogenesis and progression of heart failure, regardless of the diseases that may lead to it [23,24]. Proinflammatory cytokines contribute to LV remodeling and CHF progression, worsening its contractile function and stimulating the development of LVH, the apoptosis of cardiomyocytes, and fibrosis [25,26,27]. Patients with various diseases and pathological processes (in particular, aortic stenosis, hypertrophic cardiomyopathy, heart transplantation) and preserved LVEF are characterized by increased levels of proinflammatory cytokines identified in the myocardium and systemic circulation [28,29].

In the present study, we evaluated the gender peculiarities of immune inflammation laboratory markers in EH complicated by CHF with preserved LVEF. In general, among the patients with hypertension combined with CHF with preserved LVEF, the level of proinflammatory status indicators (CRP, TNF-α, IL-6) corresponded to the normative range of values; however, it was significantly higher in males than in females. The power of associations of these parameters with systolic and diastolic BP magnitudes, the concentric pattern of left ventricular hypertrophy, left atrium dilatation, LV diastolic dysfunction, and the level of exercise tolerance seems to confirm the concept of subclinical inflammation contribution to pathogenesis and hypertension and CHF with preserved LVEF. Of course, we should pay special attention to the specificity of comorbid factors in our sample, which predetermines a high probability of revealing the intensity of the proinflammatory pattern. Thus, all patients characterized by a mild–moderate increase in blood pressure were verified in office settings. The majority of males and females had abdominal obesity, and some of the examined individuals had type 2 diabetes or subclinical hyperuricemia. More than half of the participants possessed overt metabolic syndrome. In all subjects, clinically nonsevere heart failure with preserved LV contractility was verified. Each of these clinical features of the sample involved the participation of proinflammatory factors in the structural and functional damage of the cardiovascular system. Under the alliance of these diseases, as a rule, low-intensity inflammation, oxidative stress, lipid, carbohydrate, purine metabolism disorders, endothelial dysfunction, hyperactivity of neurohormonal regulatory systems (sympathetic–adrenal, renin–angiotensin–aldosterone) not only coexist but also are in reciprocal conflicting relations. The importance of metabolic syndrome within the framework of the discussed issue should be especially emphasized. Obesity is considered a risk factor for such diseases and conditions as insulin resistance, type 2 diabetes mellitus, and atherosclerosis. Visceral adipose tissue serves as a source of systemic excess of inflammatory autacoids. Excess of intra-abdominal and intrapericardial adipose tissue, documented by imaging techniques, to a certain extent, is associated with diastolic LV dysfunction through a proinflammatory pattern [30]. As a rule, markers of inflammation are closely associated with the amount of retroperitoneal but not subcutaneous fat [31]. Clearly, the data presented are new with regards to the sex differences of the parameters of inflammatory mediators’ systemic action in the sample of patients with a particular form of CHF. At the same time, the pathogenic role of CRP and proinflammatory cytokines (TNF-α, IL-1β, IL-6) overexpression is well known in the maintenance of arterial hypertension, as well as in the development of cardiac and vessel remodeling, renal dysfunction, impairment of the contractile and relaxing myocardial ability, tolerance to exercise, and progression of heart failure [9,32,33,34]. The data established in the current research indicate the similarity of the relationship between CRP, TNF-α, and IL-6 with structural and functional parameters of the left cardiac chambers in males and females with EH complicated by CHF with preserved LVEF. The presence of metabolic syndrome in patients with hypertension and heart failure, as a rule, augments the association of inflammatory phenotype degree with the damage of target organs. Diastolic LV dysfunction, often recorded in CHF with preserved LVEF, serves as a traditional satellite of a moderate increase of proinflammatory cytokine content in serum and myocardial tissue, to a certain extent, indicating their pathogenic relationship. This provides additional foundations to interpret metabolic syndrome as a result of chronic conflict between local inflammatory and systemic neuroendocrine regulatory mechanisms, increasing the values and decreasing the effectiveness of body defense [5].

Some previous studies pointed out that diastolic LV dysfunction is more closely related to the proinflammatory pattern than the hypertrophy of this heart chamber [35]. Our findings revealed a similar fact with reference to the male group but not to the female group. Indeed, based on the values of the coefficient r, in males with EH complicated by CHF with preserved LVEF, the CRP, TNF-α, and IL-6 levels correlated more closely with LAVI, E/e’, and e’ than with LVMI.

Impairment of diastolic LV function is associated with an increase in the rate of fibrous tissue formation. The change in the balance between pro- and antifibrotic factors is associated with the synthesis of the regulation disorders of collagen chains from their precursors, procollagen types I and III. The main profibrotic bioregulators are synthesized by fibroblasts and cardiomyocytes (components of the renin–angiotensin–aldosterone system, interleukin-6, and tumor necrosis factors α and β) and by immune cells of the blood, such as macrophages and other leukocytes as well (cytokines, growth factors, galectin-3) [36,37]. In other words, in diastolic dysfunction pathogenesis, the acceleration of the rates of intracardiac fibrous tissue formation stimulated by proinflammatory cytokines is important. Local intracardiac inflammation accompanying the expression of profibrotic factors is considered one of the mechanisms of contractile and relaxing dysfunction and remodeling of the myocardium (in particular, in arterial hypertension and CHF).

Our findings demonstrated the linear interrelation of inflammatory autacoids systemic blood levels and diastolic LV function disorders. These data are largely comparable to the results of several other studies. Thus, Wu et al. reported the relationship between the concentrations of TNF-α and IL-6 in the blood and indices of tissue Doppler-graphic parameters of LV diastolic dysfunction in patients with CHF with preserved EF. The authors found a strong correlation of these cytokines with the E/e’ ratio (positive) and e’ (negative) [29]. It has been estimated that the ability of cardiomyocytes to synthesize TNF-α is proportional to the degree of diastolic tension. Our data showed that the serum contents of CRP, TNF-α, and IL-6 in patients with EH complicated by CHF with preserved LVEF are clearly associated with the severity of myocardial diastolic dysfunction regardless of the patient’s sex, but the strength of the identified correlative interrelationships is greater among the surveyed male patients.

## 5. Conclusions

1. As compared to females, male patients with chronic essential arterial hypertension complicated by CHF with preserved left ventricular ejection fraction were characterized by significantly higher blood levels of several proinflammatory autacoids, such as C-reactive protein, tumor necrosis factor alpha, and interleukin-6. However, these levels were within normal ranges, hence the systemic inflammatory response was at a subclinical degree of severity.

2. Arterial hypertension complicated by CHF with preserved left ventricular ejection fraction, regardless of the patient’s sex, is associated with a significant association between blood levels of C-reactive protein, tumor necrosis factor alpha, and interleukin-6 on the one hand, and indicators characterizing the degree of arterial hypertension, exercise tolerance, left ventricular hypertrophy, diastolic dysfunction, and dilatation of the left atrium on the other hand. All these correlations were stronger among male patients.

3. Male patients with essential arterial hypertension complicated by CHF with preserved left ventricular ejection fraction, in contrast to females, have a closer correlation with the studied proinflammatory status markers with those indicators characterizing the degree of diastolic left ventricle dysfunction, rather than those indicators reflecting the magnitude of its hypertrophy.

Limitations of the research: These can be associated with the analytical presentation of data without taking into account the stratification of participants by the sign of being in menopause (for females), the degree of arterial hypertension, the degree of diastolic dysfunction, and the values of NT-proBNP.

## Figures and Tables

**Table 1:** The baseline characteristics of the examined patients with EH complicated by CHF with preserved LV systolic function (M ± std. d.).

Parameters	Males (n = 55)	Females (n = 49)	*p* Value
Age, years	53.7 ± 6.4	51.5 ± 6.2	0.19
BMI, kg/m^2^	33.6 ± 5.2	30.3 ± 3.6	0.12
HbA1c, %	6.08 ± 0.09	5.92 ± 0.08	0.39
NT-proBNP, pg/mL	287 ± 96.9	282 ± 105	0.84
The 6 min walk test, m	407 ± 74	420 ± 80	0.14
Office SBP, mmHg	156 ± 8.8	148 ± 6.4	0.18
Office DBP, mmHg	90.2 ± 4.4	85.5 ± 5.7	0.32
Heart rate, bpm	74.3 ± 12.5	76.1 ± 8.7	0.21
Ejection fraction, %	62.8 ± 0.68	61.6 ± 0.73	0.27
LVMI, g/m^2^	128 ± 1.52	121 ± 1.17	0.03
LAVI, mL/m^2^	36.7 ± 0.36	36.1 ± 0.34	0.24
E/e’	9.85 ± 0.40	8.63 ± 0.38	0.03
e’ averaged, cm/s	7.41 ± 0.23	8.31 ± 0.24	0.009
CRP, mg/L	4.91 ± 0.15	3.77 ± 0.17	0.001
TNF-α, pg/mL	5.81 ± 0.24	4.74 ± 0.29	0.002
IL-6, pg/mL	5.52 ± 0.29	4.21 ± 0.22	0.001

Abbreviations: BMI: body mass index, HbA1c: glycosylated hemoglobin, NT-proBNP: N-terminal brain natriuretic peptide, SBP: systolic blood pressure, DBP: diastolic blood pressure, LVMI: left ventricular mass index, LAVI: left atrium volume index, CRP: C-reactive protein, TNF-α: tumor necrosis factor alpha, IL-6: interleukin-6.

**Table 2:** Significant correlations of C-reactive blood protein level (mg/L) with other laboratory and instrumental indicators of cardiovascular prognosis in males and females with hypertension, complicated by chronic heart failure with preserved left ventricular ejection fraction.

Parameters	Males (n = 55)		Females (n = 49)	
	r Coefficient	*p*-Value	r Coefficient	*p*-Value
LVMI, g/m^2^	0.600	<0.001	0.603	<0.001
RWT	0.405	<0.001	0.415	<0.001
LAVI, mL/m^2^	0.728	<0.001	0.567	<0.001
e’, cm/s	−0.725	<0.001	−0.441	<0.001
E/e’	0.641	<0.001	0.417	<0.001
Office SBP, mmHg	0.655	<0.001	0.535	<0.001
Office DBP, mmHg	0.470	<0.001	0.415	<0.001
6 min distance, m	−0.568	<0.001	−0.610	<0.001

Abbreviations: LVMI: left ventricular mass index, LAVI: left atrium volume index, RWT: relative wall thickness, SBP: systolic blood pressure, DBP: diastolic blood pressure.

**Table 3:** Significant correlations of interleukin-6 blood level with other laboratory and instrumental indicators of cardiovascular prognosis in males and females with hypertension complicated by CHF with preserved left ventricle ejection fraction.

Parameters	Males (n = 55)		Females (n = 49)	
	r Coefficient	*p*-Value	r Coefficient	*p*-Value
LVMI, g/m^2^	0.538	<0.001	0.393	<0.001
RWT	0.442	<0.001	0.323	0.003
LAVI, mL/m^2^	0.653	<0.001	0.457	<0.001
e’, cm/s	−0.754	<0.001	−0.522	<0.001
E/e’	0.719	<0.001	0.393	<0.001
Office SBP, mmHg	0.746	<0.001	0.431	<0.001
Office DBP, mmHg	0.598	<0.001	0.333	0.003
6 min distance, m	−0.617	<0.001	−0.373	0.001

Abbreviations: LVMI: left ventricular mass index, LAVI: left atrium volume index, RWT: relative wall thickness, SBP: systolic blood pressure, DBP: diastolic blood pressure.

**Table 4:** Significant correlative relationships of tumor necrosis factor alpha level with other laboratory and instrumental indicators of cardiovascular prognosis in males and females with hypertension, complicated by CHF with preserved left ventricle ejection fraction.

Parameters	Males (n = 55)		Females (n = 49)	
	r Coefficient	*p*-Value	r Coefficient	*p*-Value
LVMI, g/m^2^	0.596	<0.001	0.569	<0.001
RWT	0.523	<0.001	0.510	<0.001
LAVI, mL/m^2^	0.665	<0.001	0.606	<0.001
e’, cm/s	−0.709	<0.001	−0.553	<0.001
E/e’	0.636	<0.001	0.450	<0.001
Office SBP, mmHg	0.670	<0.001	0.596	<0.001
Office DBP, mmHg	0.532	<0.001	0.520	<0.001
6 min distance, m	−0.640	<0.001	−0.513	<0.001
